# Performance of random forests and logic regression methods using mini-exome sequence data

**DOI:** 10.1186/1753-6561-5-S9-S104

**Published:** 2011-11-29

**Authors:** Yoonhee Kim, Qing Li, Cheryl D Cropp, Heejong Sung, Juanliang Cai, Claire L Simpson, Brian Perry, Abhijit Dasgupta, James D Malley, Alexander F Wilson, Joan E Bailey-Wilson

**Affiliations:** 1 Inherited Disease Research Branch, National Human Genome Research Institute, National Institutes of Health, Baltimore, MD 21224, USA; 2 Clinical Sciences Section, National Institute of Arthritis and Musculoskeletal and Skin Disease, National Institutes of Health, Bethesda, MD 20892, USA; 3 Center for Information Technology, National Institutes of Health, Bethesda, MD 20892, USA

## Abstract

Machine learning approaches are an attractive option for analyzing large-scale data to detect genetic variants that contribute to variation of a quantitative trait, without requiring specific distributional assumptions. We evaluate two machine learning methods, random forests and logic regression, and compare them to standard simple univariate linear regression, using the Genetic Analysis Workshop 17 mini-exome data. We also apply these methods after collapsing multiple rare variants within genes and within gene pathways. Linear regression and the random forest method performed better when rare variants were collapsed based on genes or gene pathways than when each variant was analyzed separately. Logic regression performed better when rare variants were collapsed based on genes rather than on pathways.

## Background

The common disease/common variant hypothesis has been successful at detecting some small to moderate genetic effects for complex traits in genome-wide association studies, although a substantial proportion of the heritability remains unexplained. The paradigm of common disease/rare variant contributions to the remaining genetic variation is now of interest. New sequencing technologies have made it feasible to determine DNA sequence variations in large numbers of subjects.

Machine learning approaches are attractive in terms of handling large-scale data without requiring specific distributional assumptions and are useful for detecting interaction effects of multiple predictors on a trait. The random forest (RF) method and logic regression (LR) are two machine learning methods [1]. The RF method [2] has been used in genome-wide association studies to reduce the number of genetic variants that will be used for follow-up studies and to detect variants of moderate effect in the presence of larger effect interactions [3].

In the RF method, the goal is to build a forest of models, or trees, that, when combined, explain the variation of a trait. The RF method uses a random subset of the data and a random subset of the predictors to build a tree that minimizes the out-of-bag (OOB) mean-square error (MSE) in a bootstrap sample. The samples not used to build the tree are called the OOB sample, and they are used to calculate the OOB MSE. At each node in each tree, the RF method makes a random draw from the list of predictor variables. Predictors used or selected in one node may reappear in any other node. The RF method then repeats this process using different random samples of predictors and data to produce a large collection, or forest, of trees. A variable importance score is computed for each predictor in a tree, using permutation to measure how much the OOB MSE for a given tree would increase if this predictor was randomized (made noisy). This is one method among several that can be used to evaluate the predictiveness of the predictors in the tree. The RF method then ranks the predictors based on an average, across all trees, of the variable importance scores. To prevent overfitting, the RF method sets aside OOB samples and calculates the error rate in prediction for its proposed models in each tree using the OOB sample. The RF method does not pick a final model that contains only a subset of the original large number of predictors. The top-ranked predictors in an RF model are considered to be those that are most likely to be truly causal. Users decide the cutoff that determines what proportion of the top-ranked predictors are to be used in follow-up investigations (see [1,2,4]).

LR is a model-searching method embedded in a regression framework. It uses Boolean operators to construct a flexible network of markers as a model and uses simulated annealing to control the model’s search across all variants [1,5]. LR searches all Boolean combinations of the predictor variables and finds an optimal regression model with only a handful of the original predictors. The optimal regression model is determined by a measure of model fitness to the data (MSE for linear regression). The maximum size of the final model is predetermined by the analyst, and all the predictors are equally important. To prevent overfitting, users can choose either the traditional permutation and cross-validation method or the conditional permutation test, a unique feature of LR [1,5,6].

We evaluate the performance of the RF method, LR, and simple univariate linear regression (ULR) using the Genetic Analysis Workshop 17 (GAW17) mini-exome sequence data. We want to determine whether these methods can detect genetic effects that contribute to the variation of a quantitative trait despite sparseness in the data resulting from low minor allele frequency (MAF) at many genetic variants. Neither the RF method nor LR performs tests of hypotheses in a frequentist framework. Thus power and type I error calculations under the null hypothesis are not relevant for these methods. However, the percentage of simulated data replicates in which either top-ranked predictors in the RF method or final models in LR contain true causal variants (CVs), termed the percentage of replicates (PoR), can be interpreted as a surrogate of power. Alternatively, the percentage of CVs or noncausal variants (NCVs) that are contained in the top-ranked predictors in the RF method, the final models in LR, or the most significant set of tests in ULR for multiple replicates of the simulated data can be used to evaluate whether CVs are more likely to be ranked more highly than NCVs as predictors of Q2. For LR, permutation testing is used to avoid overfitting, and it provides the PoR in which the MSE of the final model is smaller than the MSEs of the models from randomly permuted data sets.

We apply the RF and LR approaches to the simulated data after collapsing multiple rare variants (RVs) within genes and within gene pathways. Gene pathways are a set of genes that interact to perform a particular aggregate function within a cell. We use biological knowledge of gene pathways to collapse multiple RVs into single predictors.

## Methods

### Data

GAW17 provided mini-exome data [7]. We limited our machine learning methods to the Asian cohort, the largest, most homogeneous group (13,251 variants after excluding monomorphic variants in 321 individuals). We used trait Q2 after adjusting for sex, age, and smoking. We used the method of Li and Leal [8] see also [9] to recode the genotype data in two ways. First, by creating an indicator variable for the presence of at least one RV within a gene and using it to replace the genotypes of all RVs within the gene (but not collapsing the common variants), we created a gene-collapsed data set by collapsing RVs (MAF < 0.01) within genes. This resulted in 6,882 gene-collapsed variants (2,133 RV indicator variables and 4,749 common variants in 2,889 genes). Second, for genes belonging to the same pathway, we created a pathway-collapsed data set by using an indicator function for the presence of any RV (MAF < 0.01) in the same pathway. Pathways from the Gene Set Enrichment Analysis database [10] generated 2,416 pathway-collapsed variants. Common variants were excluded, and genes not belonging to a pathway were assigned their own pathway. We analyzed uncollapsed, gene-collapsed, and pathway-collapsed data in 200 replicates. After designing and beginning our analyses, we requested the trait simulation model from the GAW17 organizers so that we could find out which genetic variants contributed to Q2 (36 CVs, 13 causal genes, and 167 causal pathways in Asians).

### Simple univariate linear regression

The uncollapsed, gene-collapsed, and pathway-collapsed data were analyzed with ULR using a trend test with PLINK [11] and R after adjusting Q2 for age, smoking, and sex and after coding genetic variants as the number of minor alleles. Significance was evaluated at Bonferroni-corrected *p*-values of 0.05 based on the number of variants, genes, or pathways. The tests were ranked by ascending *p*-values after dropping any NCV that exhibited a correlation greater than 0.6 with any CV (termed an “uncorrelated NCV” [UNCV]). The percentage of both CVs and UNCVs that were ranked in the most significant 10% of tests in at least 5%, 10% and 20% of replicates was calculated. This was repeated for gene-collapsed and pathway-collapsed variants.

### Random forest method

We used Random Jungle, version 1.0.359 [12], which is a version of the RF method that has many options and functions that are distinct from the original RF implementation [2] in R. We coded the collapsed RV predictors as presence or absence of an RV, and we coded all common variant genotypes as binary predictors using dominant and recessive coding. Each RF model fitted 10,000 regression trees (required for stability when predictors have weak effects on the trait) with a minimum terminal node size of 50 observations and random sets of 7,000, 3,500, and 1,200 independent variables at each node in each tree (determined from one data replicate as suggested by Goldstein et al. [13]) for the uncollapsed, gene-collapsed, and pathway-collapsed data, respectively. After building each forest, predictors with negative importance scores were removed and a new forest was built with the remaining predictors. This process was repeated until only 10% of the original predictors remained to build the final forest.

In this study, the main purpose of RF analysis was to reduce the number of variables for further follow-up association analysis based on the rated lists of variables [3]. To evaluate its performance, we determined the PoR in which each “causal” variant, gene, and gene pathway occurred in the top 1%, 5%, and 10% of variables ranked by permutation importance scores. For these same top-ranked percentiles, we calculated the PoR with at least one causal variant, gene, or gene pathway ranked at these levels. We also calculated the percentage of CVs and UNCVs that were ranked in the top 10% of predictors in 5%, 10%, and 20% replicates and repeated this for gene-collapsed and pathway-collapsed variants.

### Logic regression

We used the LogicReg, version 1.4.8, package in R. Because of software limitations (maximum 6,000 predictors/run), we separated the analysis by chromosome. Because LR accepts only binary predictors, we coded the collapsed RV predictors as binary predictors and recoded the uncollapsed common variant genotypes as binary predictors using dominant and recessive coding. Each logic tree is composed of multiple binary predictors (leaves) connected by Boolean operators. To accommodate the situation of many causal RVs, we fitted the tree using only the “or” Boolean operator instead of the “and” operator because allowing both in the presence of many rare alleles would lead to model-fitting failures. Multiple logic trees are included in LR as additive linear terms.

For gene-collapsed data, we analyzed (1) the RVs and common variants together and (2) only the RVs. To evaluate the effect of model size, we fitted the LR using 1 tree with 3 leaves and 1 tree with 10 leaves for the gene-collapsed data with combined rare and common variants, and for the gene-collapsed data with only RVs we fitted the LR using either 1 tree with 10 leaves or 3 trees with 10 leaves. To demonstrate that overfitting might be a problem for this data set, we conducted permutation tests for the gene-collapsed 3-trees-10-leaves procedure on three chromosomes with CVs (2, 3, 6) as well as three chromosomes without any CVs (1, 4, 5) by permuting the trait values and refitting at the same model size. For each chromosome we evaluated whether the MSE for the real data was less than the observed MSE values for 25 permuted data sets.

For the pathway analysis, we fitted models using 2 trees with 20 leaves. For each predictor, we calculated the PoR that identified the predictor in the fitted model, denoted as the pick rate [6]. Although power does not apply to LR, one can consider the pick rate of the causal markers as a surrogate.

We also calculated the percentage of CVs and UNCVs that were included in the fitted model in 5%, 10%, and 20% of replicates for the gene-collapsed and pathway-collapsed variants. For each analysis, we also calculated the MSE for the optimal model in each replicate, averaged this MSE across replicates, and compared this average MSE for chromosomes with CVs versus chromosomes without CVs.

## Results

### Simple univariate linear regression

Additional table 1 (see additional file 1 for additional table 1) shows that for the ULR on uncollapsed data, only three CVs were significantly associated with Q2 at a Bonferroni-corrected *p*-value of 0.05 in one replicate each (PoR = 0.5%). However, 56 UNCVs were detected at this Bonferroni-corrected *p*-value (once for each UNCV) across the 200 replicates. In the gene-collapsed data, 2 out of 13 causal genes and 62 out of 2,876 noncausal genes were detected at a Bonferroni-corrected *p*-value of 0.05. For pathway-collapsed data, 17 out of 167 causal pathways and 47 out of 2,249 noncausal pathways were detected. Table 1 shows that a higher proportion of CVs than UNCVs had ULR *p*-values that were ranked in the most significant 10%, with similar findings for gene-collapsed and pathway-collapsed variants.

### Random forest method

For the uncollapsed data for the RF analysis, each CV was ranked in the top 10% of predictors in at least 5% of replicates (range 5–24%) (Additional table 1). Few CVs were ranked in the top 1% in multiple replicates. However, 34% of replicates ranked at least one CV in the top 1% of predictors, 91% of replicates ranked at least one CV in the top 5% of predictors, and all 200 replicates ranked at least one CV in the top 10% of predictors.

Additional table 2 (see additional file 2 for additional table 2) shows the results for the gene-collapsed and pathway-collapsed data. Each causal gene was ranked in the top 10% of predictors in at least 9% of replicates (range 9–40%). However, 89%, 98%, and 98% of replicates ranked at least one causal gene in the top 1%, 5%, and 10% of all 2,889 genes, respectively. Each causal gene was in at least one causal pathway that was ranked in the top 10% of the 2,416 pathways in at least 6% of replicates (range 6–66%). Five pathways containing CVs were ranked in the top 10% of pathways in at least 50% of replicates. However, 100% of replicates ranked at least one causal pathway in the top 1% of all 2,416 pathways.

Table 1 shows that for uncollapsed, gene-collapsed, and pathway-collapsed variants, a higher proportion of CVs, causal genes, or causal pathways were ranked in the top 10% of predictors in multiple replicates than was observed for their uncorrelated noncausal analogs.

**Table 1:** Percentage of CVs and UNCVs that were in the top-ranked 10% of predictors (RF and ULR) or that were included in the final model (LR) in at least 5%, 10%, and 20% of the 200 simulated replicates

Data set	Total number^a^		Random forest, % variants ranked in the top 10% of predictors			Univariate linear regression, % variants ranked in the most significant 10%			Logic regression, % variants included in final model		
		
			In ≥5 PoR	In ≥10 PoR	In ≥20 PoR	In ≥5 PoR	In ≥10 PoR	In ≥20 PoR	In ≥5 PoR	In ≥10 PoR	In ≥20 PoR
Uncollapsed	CVs	36	100	72	33	94	72	39			
	
	UNCVs	12,485	76	46	8	90	25	3			

Gene-collapsed	CVs	15	100	100	50	87	73	53	91^b^	64	45
	
	UNCVs	6,642	98	81	24	90	26	3	63	32	9

Pathway-collapsed	CVs	167	99	95	72	93	50	26	1^c^	0	0
	
	UNCVs	2,249	91	68	38	88	32	8	0	0	0

UNCVs are noncausal variants that were not used in the simulation to determine Q2, were not in one of the causal genes, and did not display correlation of at least 0.6 with any CVs.

^a^ Total number of nonmonomorphic variants in Asians. For LR we excluded 4 common CVs and 4,725 common NCVs.

^b^ Final LR model: 3 trees with 10 leaves.

^c^ Final LR model: 2 trees with 20 leaves.

### Logic regression

We evaluated LR under different settings by changing the input predictors and the prefixed model size and structure. When both common variants and gene-collapsed variants were used as inputs, the pick rates of the CVs were low; the causal genes were included in the optimal model in a maximum 22% of replicates. When only the collapsed RVs were included (Additional table 2), the 3-trees-10-leaves modeling procedure led to a better pick rate than the 1-tree-10-leaves procedure, because it allowed different estimates for effect sizes of the logic trees. For the 3-trees-10-leaves model, 97% of replicates produced at least one optimal model containing a true CV (out of the 22 chromosomal models per replicate).

**Figure 1:**
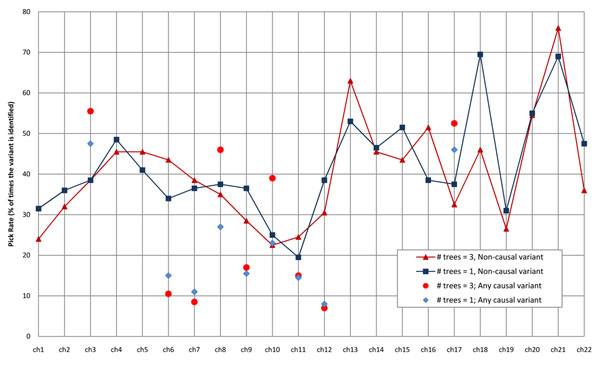
LR pick rate (by chromosome) for the most frequently identified noncausal genes and any causal gene using gene-collapsed data

In Figure 1, for each chromosome for the gene-collapsed data, we plot the pick rates for the most frequently identified NCVs as lines and CVs as dots. Despite strong spurious associations with NCVs, the 3-trees-10-leaves model procedure led to the highest pick rates for any CV on chromosomes 3, 8, 10, and 17, showing that the proper model-fitting procedure has the potential to identify true associations.

The MSE of the optimal model on each chromosome averaged over all replicates was usually slightly lower for chromosomes containing CVs compared to chromosomes without CVs. However, the permutation test for the gene-collapsed data on three chromosomes with CVs and three chromosomes without CVs showed that the MSE for the optimal model generally was not less than the MSEs of the permuted data sets, regardless of whether or not the chromosome contained CVs, indicating little ability to separate effects of true CVs from noise in this data set.

Additional table 2 (see additional file 2) shows that in the pathway-collapsed analyses, the pathways containing causal genes were rarely included in the optimal models. Table 1 shows that for the gene-collapsed analyses, a higher percentage of causal genes than uncorrelated noncausal genes are included in the final models in multiple replicates, but it also illustrates that the pathway-collapsed analyses exhibit virtually no replication of results across the different replicates.

## Discussion

The simulated quantitative trait Q2 was caused by 36 variants with small individual effects on the variation of the trait [7]. One CV had an average locus-specific heritability (*h*^2^) [14] (across 200 replicates) of 0.04, five CVs had locus-specific *h*^2^ between 0.011 and 0.017, and the remaining CVs had locus-specific *h*^2^ < 0.01. Thus there was little power to detect CVs in this small sample using any statistical method. ULR of uncollapsed data detected only 3 of the CVs in a single replicate each (PoR = 0.5%) and 56 false-positive results at a Bonferroni-corrected *p*-value of 0.05. This false-positive rate is inflated over the expected rate of 5 per 100 genome-wide analyses. This inflation may be due to only one set of genotypes being used in all 200 replicates, the multicollinearity of sequence data, or other factors related to the simulations. Permutation testing should be used in real data to control type I error. The collapsed analyses had better power and more appropriate false-positive rates.

For the RF method using the uncollapsed data, 91% and 100% of replicates ranked at least one CV in the top 5% and 10% of predictors, respectively. In the gene-collapsed and pathway-collapsed data, 89% and 100% of replicates ranked at least one causal gene or pathway in the top 1% of predictors, respectively. CVs were detected more often in gene-collapsed and pathway-collapsed analyses than in uncollapsed analyses. However, individual CVs, genes, and pathways were not consistently ranked among the top 10% of predictors across the 200 replicates, and of course many NCVs exist in even the top 1% of predictors. However, a higher proportion of CVs, causal genes, and causal pathways were ranked among the top 10% of predictors in multiple replicates (Table 1) than were the comparable noncausal variants, genes, or pathways.

If we consider that the main function of the RF method is to reduce the large number of markers to a set of candidates for inclusion in additional studies, then these results suggest that for polygenic models with small locus-specific *h*^2^, such as the one simulated here, larger samples would be desirable and, in a similarly small study, at least the top 5–10% of predictors for uncollapsed data and the top 1% of predictors in gene-collapsed or pathway-collapsed analyses should be used in follow-up studies to give a reasonably high probability that at least one causal locus will be selected.

We observed better performance for LR in the gene-collapsed data than in the pathway-collapsed data. The performance of LR can vary substantially depending on the prefixed model size and structures and the input predictors. Models with multiple linear terms of logic trees (multiple trees) appear more appropriate for RVs with large variance in effect size. In the gene-collapsed data, even with small effect sizes of CVs on Q2, higher proportions of causal genes than noncausal genes were included in the fitted models across multiple replicates. Future extensions to LR software that incorporate all predictors across the genome in a single analysis may improve performance.

No interactions between loci were simulated for Q2. However, in reality, genetic elements can work both collectively and separately. Thus machine learning methods may be especially useful in such situations. Finding networks or motifs in the genetic material is an unsolved problem in the literature.

## Conclusions

We applied the RF, LR, and ULR approaches to a simulated polygenic quantitative trait and sequence data with both common and rare variants. ULR had low power to detect any CVs in this small sample. All three methods ranked larger proportions of CVs than NCVs among the top-ranked predictors in multiple replicates. When using the RF method or LR, we suggest that multiple trees and permutation tests be used for analysis. Our results leave open the question of whether a larger sample could improve RF and LR performance when effect sizes are small. Although not providing tests of hypotheses, machine learning approaches such as the RF method and LR can help researchers elucidate the causes of variation in a quantitative trait by providing a putative set of candidate genes for further study.

## Competing interests

The authors declare that there is/are no competing interests.

## Authors’ contributions

YK and QL designed the study, performed the RF and LR analyses, and drafted the manuscript. CDC performed the ULR and drafted the manuscript. HS, JC, CLS, and BP performed quality control analyses and prepared the data for the analyses. AD, JDM and AFW participated in study design and critically revised the manuscript. JEBW designed the study, drafted and revised the manuscript. All authors read and approved the final manuscript..

## Supplementary Material

Additional File 1 Click here for file

Additional File 2 Click here for file
