# Supplementary material for: Performance of random forests and logic regression methods using mini-exome sequence data
Source: BMC Proc. 2011 Nov 29;5(Suppl 9):S104. doi: 10.1186/1753-6561-5-S9-S104 (PMC3287827; doi:10.1186/1753-6561-5-S9-S104)
Supplement: Additional File 1 [file 1753-6561-5-S9-S104-S1.pdf]

Additional file 1 for "Performance of random forests and logic regression methods using mini-exome sequence data"

File name: Additional_file_1_S104_Kimetal.pdf

File format: Adobe pdf format

Additional table 1 - Uncollapsed data results in the RF method and ULR of CVs for Q2

				Random forest				Univariate linear regression	
				PoR in				PoR under Bonferroni-corrected p-value	
CV	Gene	β	MAF	Top 1%	Top 5%	Top 10%	Highest rank across replicates	of 3.77×10^{-6}	Minimum p-value across replicates
C2S354	*GCKR*	0.38	0.0140	1	16	17	95	0	4.92×10^{-4}
C3S4859	*BCHE*	0.59	0.0047	1	11	14	22	0	1.15×10^{-5}
C3S4860	*BCHE*	0.25	0.0016	0	2	18	259	0	7.55×10^{-3}
C3S4862	*BCHE*	1.02	0.0016	0	5	23	176	0	2.02×10^{-3}
C3S4869	*BCHE*	1.02	0.0016	0	3	13	242	0	7.74×10^{-4}
C3S4875	*BCHE*	1.09	0.0016	0	5	17	190	0.5	2.82×10^{-6}
C3S4876	*BCHE*	0.76	0.0016	1	3	23	123	0	3.39×10^{-4}
C3S679	*RARB*	0.64	0.0078	1	7	9	130	0	6.62×10^{-4}
C6S5380	*VNN1*	0.24	0.2648	8	21	21	32	0	4.41×10^{-5}

C6S5412	*VNN3*	0.64	0.0016	0	5	22	305	0	1.54×10^{-3}
C6S5439	*VNN3*	0.10	0.0016	0	2	15	348	0	6.80×10^{-3}
C6S5441	*VNN3*	0.27	0.1713	2	9	9	28	0	6.70×10^{-4}
C7S5132	*INSIG1*	0.20	0.0016	0	4	15	381	0	4.27×10^{-3}
C7S5133	*INSIG1*	0.20	0.0016	0	4	24	448	0	1.35×10^{-2}
C7S5144	*INSIG1*	0.19	0.0016	1	2	22	118	0	2.10×10^{-3}
C8S1741	*PLAT*	0.68	0.0078	2	6	6	15	0	8.01×10^{-5}
C8S1742	*PLAT*	0.85	0.0016	0	2	16	401	0	3.28×10^{-3}
C8S1758	*PLAT*	0.93	0.0031	2	6	13	53	0	5.50×10^{-4}
C8S1772	*PLAT*	0.26	0.0031	3	3	5	15	0	8.15×10^{-3}
C8S530	*LPL*	0.73	0.0016	0	3	21	431	0	3.75×10^{-3}
C9S367	*VLDLR*	0.58	0.0016	0	4	21	247	0	1.72×10^{-2}
C9S377	*VLDLR*	1.22	0.0031	3	5	6	37	0	3.65×10^{-4}
C9S430	*VLDLR*	0.56	0.0016	0	3	13	313	0	1.60×10^{-3}
C9S497	*VLDLR*	0.66	0.0016	0	3	24	283	0	4.60×10^{-4}
C10S3048	*SIRT1*	0.83	0.0031	3	6	9	7	0	3.22×10^{-4}
C10S3050	*SIRT1*	0.97	0.0016	0	3	16	212	0.5	2.82×10^{-6}
C10S3107	*SIRT1*	0.94	0.0016	0	5	22	373	0	2.53×10^{-3}

C10S3110	*SIRT1*	0.10	0.0047	1	3	5	52	0	1.62×10^{-3}
C11S5292	*PDGFD*	0.58	0.0187	6	12	14	4	0.5	1.97×10^{-7}
C11S5299	*PDGFD*	0.82	0.0016	0	7	21	345	0	3.79×10^{-3}
C11S5302	*PDGFD*	0.82	0.0031	1	3	6	49	0	2.00×10^{-4}
C12S211	*VWF*	0.34	0.0016	0	3	17	379	0	1.84×10^{-2}
C17S1007	*SREBF1*	0.53	0.0047	2	4	6	47	0	4.46×10^{-4}
C17S1024	*SREBF1*	0.45	0.0031	3	6	9	35	0	6.93×10^{-5}
C17S1046	*SREBF1*	0.63	0.0062	2	8	11	58	0	1.76×10^{-4}
C17S1056	*SREBF1*	0.52	0.0016	0	5	24	326	0	3.88×10^{-3}
