# Supplementary material for: Performance of random forests and logic regression methods using mini-exome sequence data
Source: BMC Proc. 2011 Nov 29;5(Suppl 9):S104. doi: 10.1186/1753-6561-5-S9-S104 (PMC3287827; doi:10.1186/1753-6561-5-S9-S104)
Supplement: Additional File 2 [file 1753-6561-5-S9-S104-S2.pdf]

Additional file 2 for "Performance of random forests and logic regression methods using mini-exome sequence data"

File name: Additional_file_2_S104_Kimetal.pdf

File format: Adobe pdf format

Additional table 2 - Performance of random forest and logic regression for gene-collapsed and pathway-collapsed data

			Random forests: PoR[b]						Logic regression: pick rate[c]		
			Gene-collapsed			Pathway-collapsed			Gene-collapsed		Pathway-collapsed,
Gene	Number of CVs[a]	Number of pathways[a]	1%	5%	10%	1%	5%	10%	1 tree[d]	3 trees[e]	2 trees[f]
BCHE	6	5	4	12	24	0–7	7–23	18–32	31	39	2–12
RARB	1	15	0	3	14	0–5	5–54	6–66	21	25	1–4
VNN3	2	5	0	3	15	0–2	5–27	12–39	12	7	1–5
INSIG1	3	15	0	1	17	0–8	5–33	6–54	11	9	1–4
LPL	1	37	3	9	21	0–11	1–37	11–40	2	5	1–3
PLAT	4	33	4	7	23	0–11	5–38	13–40	26	43	1–12
VLDLR	4	21	2	5	13	0–13	2–48	8–43	16	17	1–4

SIRT1	4	9	1	6	15	0–3	5–30	11–46	23	39	1–6
PDGFD	2	7	1	6	17	2–7	13–54	22–55	15	15	1–3
VWF	1	22	1	2	24	0–13	1–54	6–55	8	7	1–3
SREBF1	4	13	8	19	40	0–13	6–47	15–45	46	53	1–14
GCKR	1*		1	7	38						
VNN1	1*		3	15	26						
VNN3	1*		2	8	14						
PDGFD	1*		5	15	27						

[a] Number of CVs in the listed genes and number of pathways including the listed gene.

[b] PoR including the listed variants in the top-ranked 10% of variables.

[c] PoR producing a fitted model including the listed variant.

[d] The final model had one tree and 10 leaves.

[e] The final model had three trees and 10 leaves.

[f] The final model had two trees and 20 leaves.
